# Dynamic Management of Lung Cancer Care During Surging COVID-19

**DOI:** 10.3389/fsurg.2021.663364

**Published:** 2021-04-07

**Authors:** Annie Wang, Stephanie H. Chang, Eric J. Kim, Jamie L. Bessich, Joshua K. Sabari, Benjamin Cooper, Travis C. Geraci, Robert J. Cerfolio

**Affiliations:** ^1^Department of Surgery, New York University Langone Health, New York, NY, United States; ^2^Department of Cardiothoracic Surgery, New York University Langone Health, New York, NY, United States; ^3^New York University Grossman School of Medicine, New York, NY, United States; ^4^Department of Medicine, New York University Langone Health, New York, NY, United States; ^5^Department of Radiation Oncology, New York University Langone Health, New York, NY, United States

**Keywords:** lung cancer, thoracic surgery, COVID-19, radiation oncology, screening

## Abstract

Management of patients with lung cancer continues to be challenging during the COVID-19 pandemic, due to the increased risk of complications in this subset of patients. During the COVID-19 surge in New York City, New York University Langone Health adopted triage strategies to help with care for lung cancer patients, with good surgical outcomes and no transmission of COVID-19 to patients or healthcare workers. Here, we will review current recommendations regarding screening and management of lung cancer patients during both a non-surge phase and surge phase of COVID-19.

## Introduction

The novel Coronavirus disease 2019 (COVID-19) is a respiratory viral infection caused by severe acute respiratory syndrome coronavirus 2 (SARS-Cov-2). Since first identification of SARS-CoV-2 in December 2019, COVID-19 emerged as a major global pandemic with over 98 million people diagnosed with COVID-19 and >2 million deaths worldwide as of January 2021 (1–3). The United States became the major epicenter for the pandemic, with 24 million patients diagnosed with COVID-19 and 408,000 deaths (1–3). Unfortunately, New York State, and in particular, New York City (NYC), has experienced the highest concentration of COVID-19 cases, with 1.2 million confirmed diagnoses and more than 41,000 deaths (1–3).

Early data, primarily from China, demonstrated that 15–20% of infected patients develop symptoms requiring hospitalization with a mortality rate of ~1–4% (4–6). Patients with underlying medical co-morbidities, particularly pulmonary disease, diabetes, obesity, and heart disease have been demonstrated to be at a higher risk for morbidity and mortality (7–9). In patients with underlying malignancies, the mortality related to concomitant COVID-19 was notably higher at ~20–40% (10–16). Lung cancer patients particularly have more severe COVID-19 disease, with an estimated mortality rate of 30–50% (11, 17, 18); this may be because lung tumor microenvironment can be modulated by SARS-CoV-2 virus, triggering a more severe cytokine response (19, 20). Lung cancer also creates immunosuppressive state that may predispose patients to COVID-19 and complications. Therefore, management of patients with lung cancer poses unique challenges during the SARS-COV2 pandemic.

COVID-19 cases continue to increase in number. While the vaccines promises to help slow the spread of COVID-19, new strains pose as another source for continued growth (21–23). It is imperative to restructure the clinical workflow and organization of hospitals to cope with the surge in the number of hospitalizations from COVID-19.

In this review article, we will discuss lung cancer management during COVID-19 pandemic and present our current institutional practice and outcomes. The work-up and management of patients depends on rate of new infections and hospitalizations for COVID-19. At NYU Langone Health (NYULH), we follow a tiered approach based on transmission rate. Transmission of COVID-19 outside of New York City (NYC), with little or no cases in NYC, and no cases at NYULH is deemed level 1. Level 2 is when there are no local transmission, but multiple cases in NYC and few NYULH cases. Transmission in NYULH is level 3, while level 4 is when NYULH surge units are full (Table 1).

**Table 1:** Triage levels used to guide management strategies.

**Triage level**	**Overall transmission**	**Local (city) transmission**	**Hospital cases**	**Hospital capacity**
Level 1	Present	Minimal/None	Minimal/None	Available beds
Level 2	Present	Multiple cases	Minimal/None	Available beds
Level 3	Present	Multiple cases	Multiple cases	Available beds
Level 4	Present	Multiple cases	Multiple cases	Surge units full

## Management During Non-surge Phase With Adequate Hospital Capacity (Level 1 and 2)

**Figure 1:**
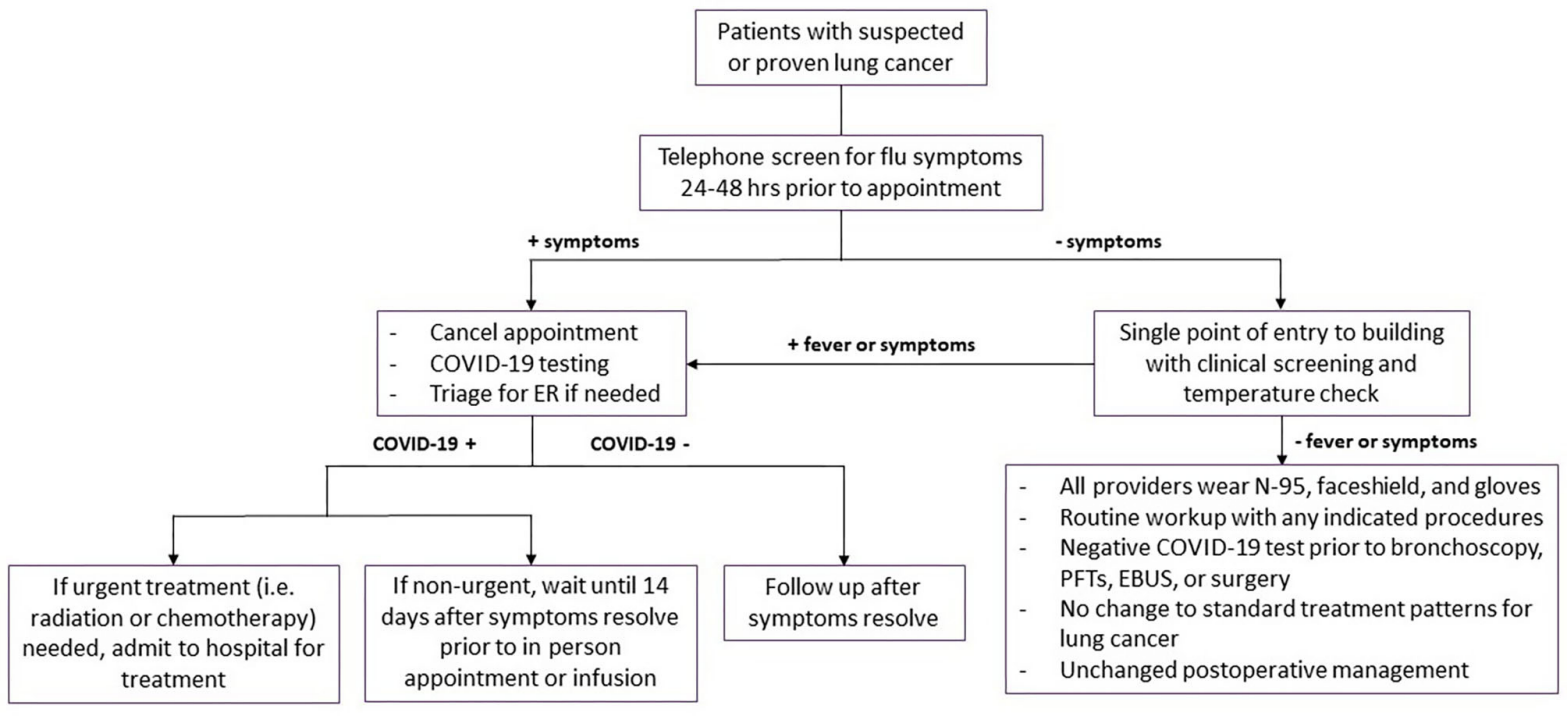
Management of patients during Level 1 and Level 2 phases of transmission. Flowchart demonstrating management and workup strategies for patients with suspected or diagnosed lung cancer during level 1 and level 2 phases of transmission. Symptoms include fevers, chills, cough, dyspnea, and loss of smell or taste. COVID-19, coronavirus disease 2019; ER, emergency room; PFTs, pulmonary function tests; EBUS, endobronchial ultrasound.

If the city and hospital are not in a surge phase of COVID-19 outbreak and have available resources, standard criteria for lung cancer work-up and management should be followed with several added precautions. Patients with lung cancer require frequent clinic visits with multiple specialists, and necessitate diagnostic work up with laboratory blood draws, imaging studies, pulmonary function tests (PFTs), bronchoscopies and transthoracic needle biopsies, radiation treatments, anti-neoplastic infusion treatments and hospital admissions for procedures. These frequent visits increased both the patient risk of exposure to COVID-19, as well as increased risk of exposure to healthcare providers taking care of lung cancer patients (Figure 1).

All patients presenting for in-person appointments should be contacted 24–48 h prior to the appointment and screened for flu-like symptoms: cough, dyspnea, fevers, chills, and loss of smell or taste. If patients have any symptoms during the telephone screening, the appointments are canceled. If patients required further evaluation of symptoms, they should be triaged for COVID-19 testing and referred to emergency room when appropriate. If patients test positive for COVID-19, in-person clinic and infusion appointments were deferred until patients were asymptomatic for a minimum of 14 days. Those requiring more immediate attention for their cancer management should be admitted for inpatient management.

All healthcare providers must wear N95 masks, eye shield, and gloves for patient contact. Face masks or coverings should be worn by all visitors and patients, regardless of their symptoms. Patient entry into the cancer center should restricted to one point of entry and access was controlled by a team of medical assistants and a nurse. COVID-19 clinical screening questions and COVID-19 test results should documented prior to registration and in-person clinic visit. If a patient screens positive with suspicion for COVID-19, the patient should be shifted to an isolation area. The patient's team would perform a clinical assessment in the isolation area and determine the appropriate action. If patients needed to be tested for COVID-19, the clinic visit and treatments were delayed until the results of the test were available.

Previously, there was concerns about bronchoscopy as a super-spreading event (24), but our published data have shown no transmission during bronchoscopy of active COVID-19 patients when appropriate precautions are taken (25). This lack of transmission has also been reproduced by other institutions (26, 27). Thus, normal preoperative bronchoscopy with biopsy or endobronchial ultrasound (EBUS) for staging should continue if patients test negative for COVID-19 within 3 days of their procedure. Similarly, PFTs should also be obtained in routine fashion if the patient has a negative test within 3 days. All treatment decisions regarding surgery, adjuvant and neoadjuvant therapy, and radiation therapy should follow normal guidelines, with negative COVID-19 testing within 3 days of any surgical procedure. All surgical procedures should be performed with operating room staff wearing N95 masks, eye shield, and gloves for patient contact. Post-operative management, including management of pneumothoraces and air leaks, should follow standard practice patterns prior to the COVID-19 pandemic.

## Management During Surge Phase or When Limited Hospital Capacity (Level 3 and 4)

### Changes to Initial Procedures for Suspected or Diagnosed Lung Cancer Patients

Patients with suspected diagnosis or newly diagnosed lung cancer require a variety of imaging modalities, diagnostic biopsies, and staging procedures. Following announcement of COVID-19 as a national emergency in March 2020, guidelines were issued by the American Association of Bronchology and Interventional Pulmonology and the American College of Chest Physicians (24), which includes the following measures to prioritize patients for diagnostic work up of known or suspected lung cancer.

**Figure 2:**
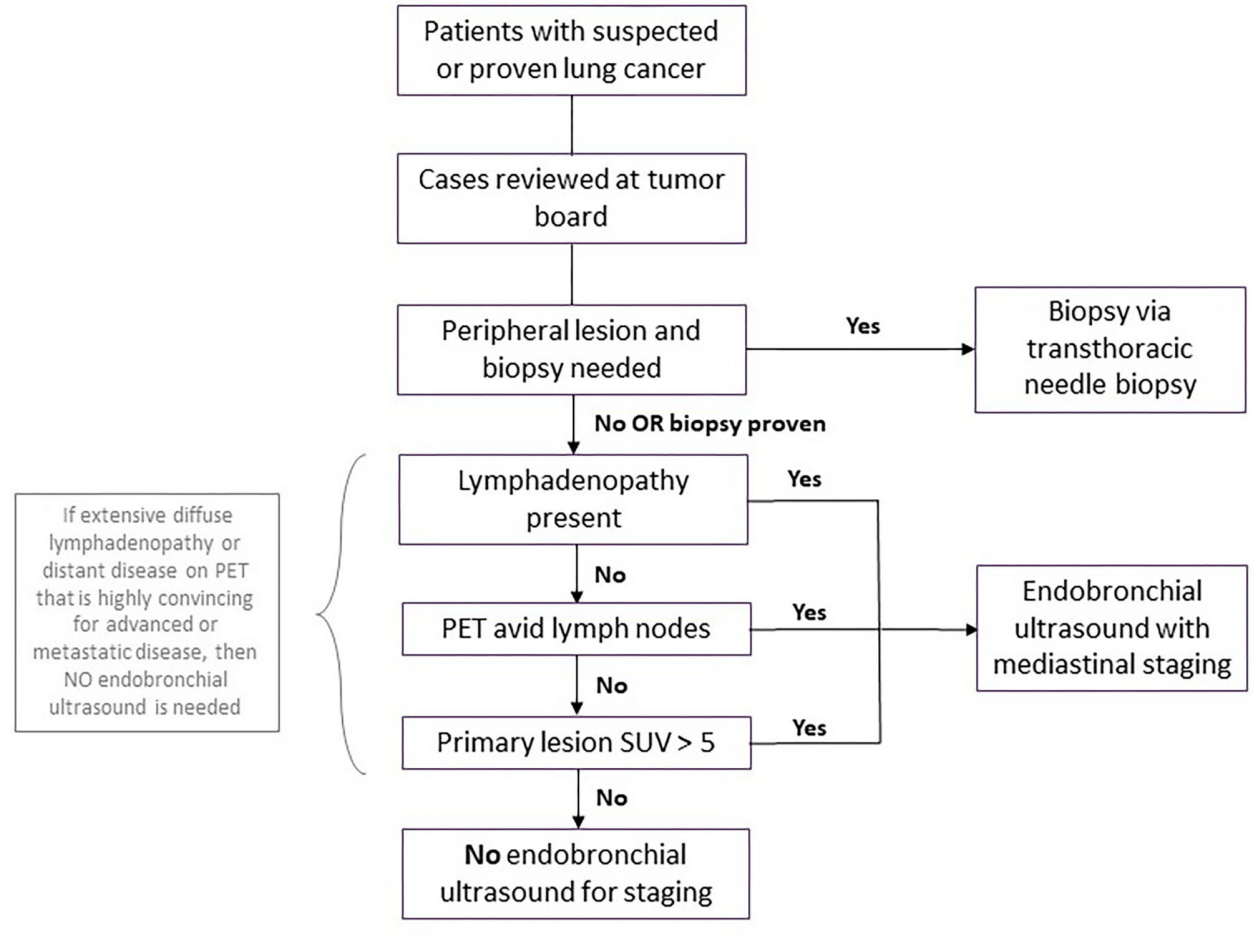
Workup for patients with suspected or proven lung cancer during surge phase or limited hospital capacity. Flowchart demonstrating workup for patients with suspected or proven lung cancer during surge phase or limited hospital capacity. A coronavirus disease 2019 (COVID-19) test should be performed prior to the biopsy or endobronchial ultrasound, with the procedure deferred for any patient with COVID-19 until the patient is negative. PET, positive electron tomography; SUV, standard uptake value.

All cases considered for bronchoscopy should be discussed at our multi-disciplinary thoracic oncology conference (MDTOC) to determine the optimal course for diagnosis and staging from the perspective of both the patient and the lung cancer team. Staging endobronchial ultrasounds were discouraged in patients without radiographic lymphadenopathy. In these patients, mediastinal and hilar staging via EBUS guided transbronchial needle aspiration (TBNA) was deferred, given the relatively low likelihood of macro- or microscopic metastases in regional lymph nodes in this setting. This was particularly valid if the standardized uptake value (SUV) of the primary lesion in the lung parenchyma was relatively low (e.g., <5–7) on positron emission tomography (PET). Conversely, patients with compelling evidence of malignant adenopathy were managed without tissue confirmation (Figure 2).

Diagnostic biopsies of peripheral lung nodules or masses in the absence of significant lymphadenopathy were performed by transthoracic needle aspiration by interventional radiology. This was considered a lower risk procedure for aerosolization of the SARS CoV-2 virus than navigational or robotic bronchoscopy for biopsy of these lesions.

For patients with confirmed peripheral lung cancer on prior biopsy who were being considered for definitive stereotactic body radiotherapy (SBRT) by radiation oncology or percutaneous cryoablation by Thoracic Radiology, we deferred staging EBUS TBNA of hilar and mediastinal nodes in those without significant adenopathy on cross sectional imaging or evidence of mediastinal or hilar FDG avidity on PET CT scan.

All patients with suspected new or recurrent diagnoses of lung cancer recommended at MDTOC to undergo EBUS TBNA should undergo SARS-CoV-2 nasopharyngeal swab RT-PCR testing prior to the procedure. Patients with negative SARS-CoV-2 testing could proceed to bronchoscopy with the interventional pulmonology team, with strict precautions within the operating room. These included full use of personal protective equipment by all OR staff (N95 masks, face shields, and impermeable gowns), as well as ensuring that only essential staff were present in the operating room for anesthesia induction and securing the airway. Patients scheduled to undergo diagnostic and staging bronchoscopy who had positive SARS-CoV-2 RT PCR testing prior to the procedure were quarantined for 2 weeks and re-tested prior to proceeding with the endoscopic procedure.

**Figure 3:**
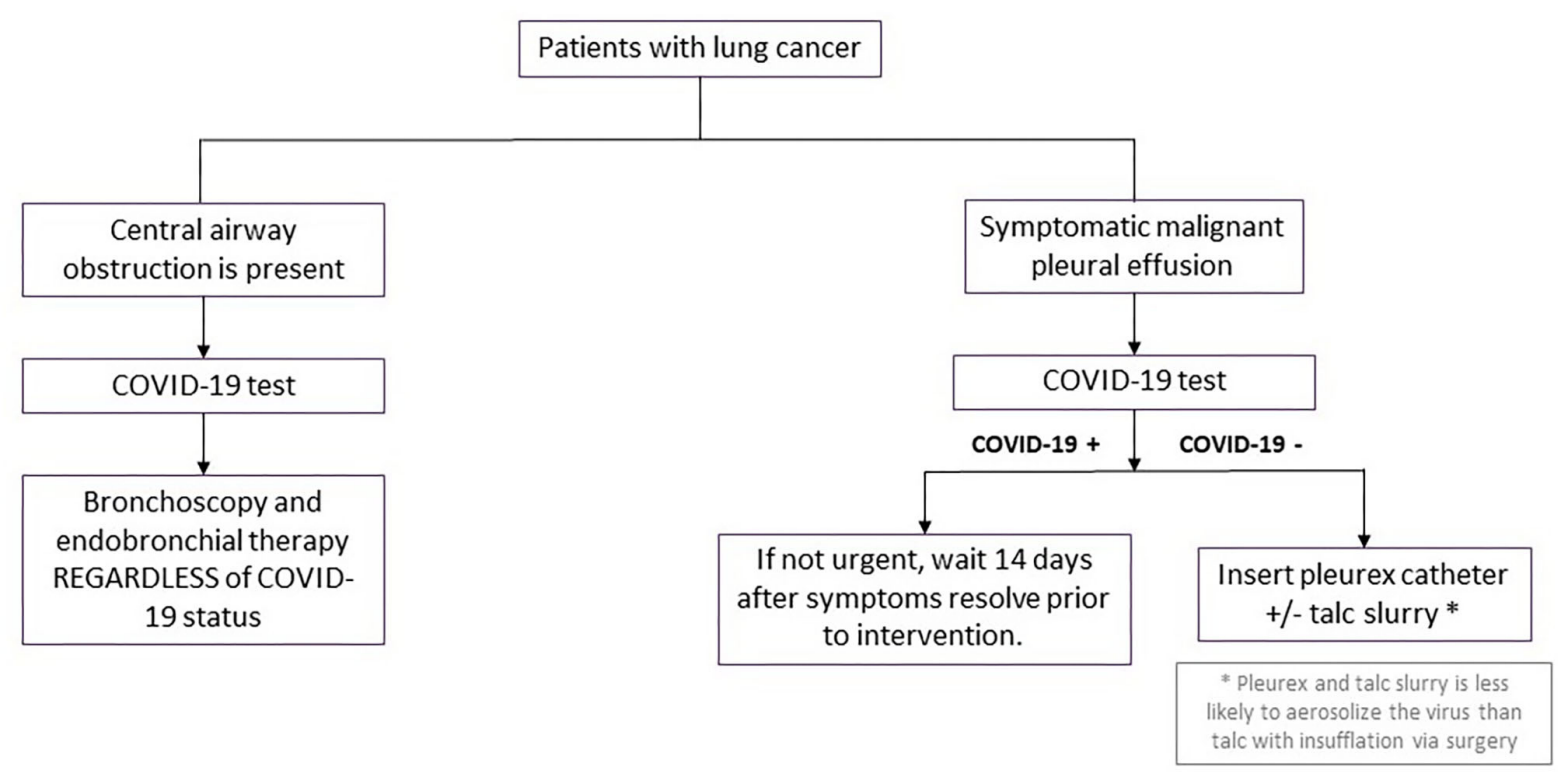
Procedural management for other scenarios in patients with lung cancer during surge phase or limited hospital capacity. Flowchart demonstrating procedural management for patients with either central airway obstruction or symptomatic malignant pleural effusions during surge phase or limited hospital capacity. COVID-19, coronavirus disease 2019.

For those lung cancer patients presenting with symptomatic central airway obstruction (persistent cough, dyspnea, hemoptysis, hypoxemia, post-obstructive atelectasis, or pneumonia), therapeutic bronchoscopy (rigid bronchoscopic debulking, thermal ablation, airway stenting) should be performed as clinically indicated regardless of SARS-CoV-2 status. SARS-CoV-2 testing should still be performed within 3 days of the planned procedure for risk stratification and preparation for safest possible conditions for the interventional pulmonologists and the OR staff (Figure 3).

We recommend continuing diagnostic and therapeutic pleural procedures in patients who are candidates for systemic anti-neoplastic therapy. Proceduralists should adhere to strict contact precautions with face shields, N95 masks, impermeable gowns, and sterile gloves. For patients symptomatic from known malignant pleural effusions, we recommend placement of indwelling pleural catheter (IPC) insertion. IPC placement is thought to be associated with lower risk of SARS-CoV-2 aerosolization than talc insufflation via video assisted thoracoscopic surgery (VATS) and could be performed in an expedited fashion on an outpatient basis. When patients had IPC insertions, a family member was remotely trained and counseled on safe and proper drainage of the IPC to obviate requirements for home visiting nurse encounters to minimize risk of COVID-19 exposure.

### Changes to Curative Surgical Management of Lung Cancer

**Figure 4:**
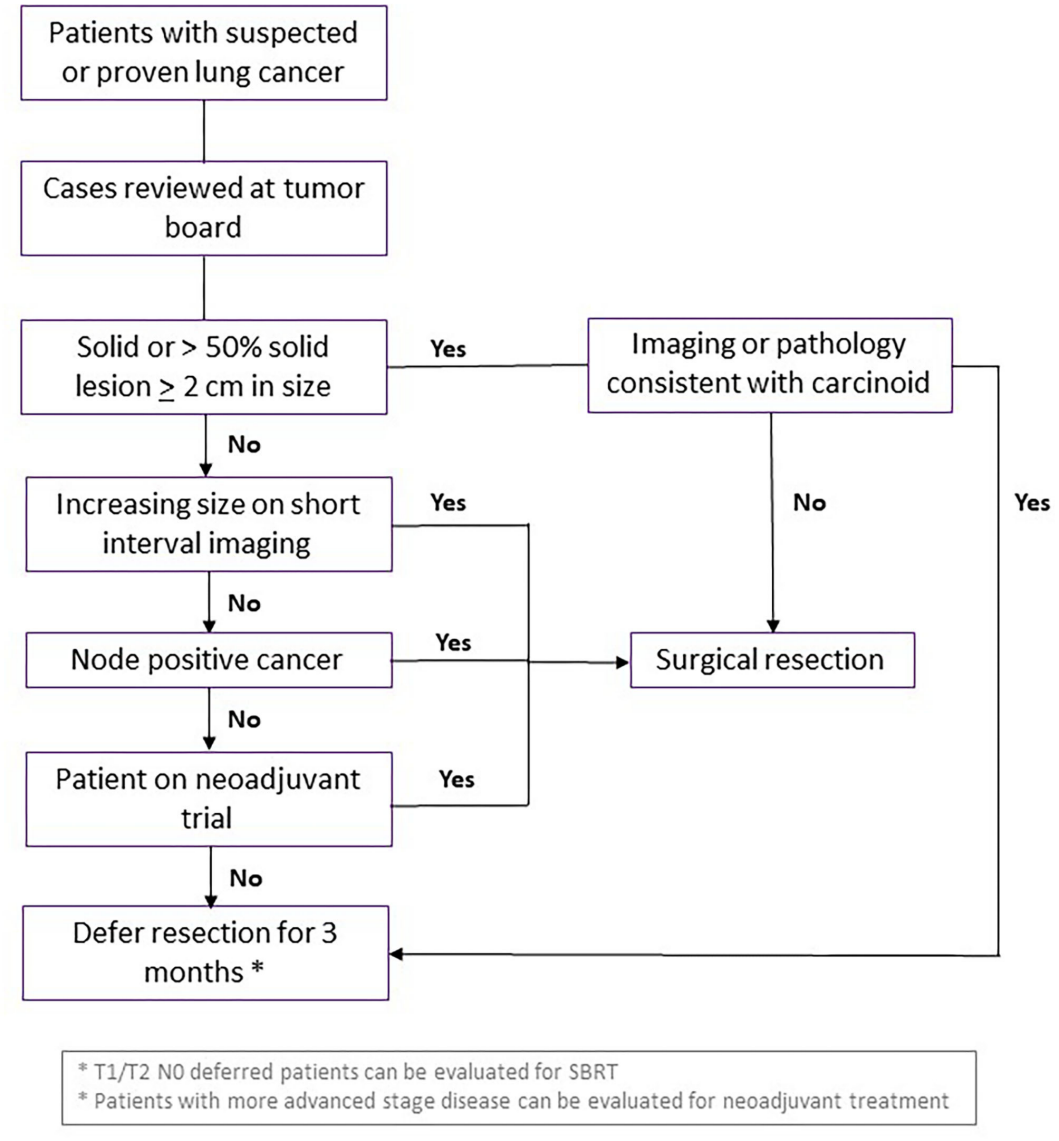
Guidelines for curative surgical management of lung cancer during surge phase or limited hospital capacity. Flowchart demonstrating criteria for patients with suspected or diagnosed lung cancer who should undergo curative surgical resection, vs. waiting or consideration for another intervention. SBRT, stereotactic body radiation therapy.

All patients should be discussed in MDTOC, with decisions regarding surgical resection determined by the growth rate and FDG avidity of the cancer in serial imaging studies, patient clinical factors and the co-morbidities including COVID-19 status. Patients with a solid or >50% solid nodule >2 cm, SUV_max_ >2.5, or change on short interval CT scan were deemed higher risk for progression of disease with delay in care and prioritized for resection. Patients on neoadjuvant trials should also undergo resection during this surge phase if there are no other contraindications. The guidelines used by our multidisciplinary group at NYULH echoed the recommendations of the American College of Surgeons and the Thoracic Surgery Outcomes Research Network (28, 29). Specifically, surgery was essentially restricted to patents whose survivorship was likely to be compromised by a surgical delay of 3 months. Conditions for early surgery included: (1) solid or predominantly solid (>50%) lung cancer or presumed lung cancer >2 cm, clinical node negative, (2) node positive lung cancer, and (3) post-induction therapy cancers. Moreover, staging to start treatment (EBUS, mediastinoscopy, diagnostic VATS) for pleural dissemination was also performed. Generally, surgery was deferred for an estimated 3 months for predominantly ground glass nodules or cancers, solid nodules or lung cancer <2 cm, and radiographic/biopsy signs of an indolent tumor (carcinoid, slowly enlarging nodule) (Figure 4).

The outcomes of these criteria applied at the peak of COVID-19 pandemic to 21 patients at NYULH were retrospectively reviewed (30), and there were no major complications, 5 minor complications and average length of stay of 2 days. Twenty-four patients deferred surgery due to patient preference and only one of these patients progressed from T3N0 to T4N0 disease. Notably, none of the nine healthcare providers involved in the surgical management of these patients contracted COVID-19.

Given the high risk to patients after lung resection, especially cancer patients that were immunocompromised and at greater risk of COVID-19 complications, surgical interventions for stage 1 NSCLC should be deferred when clinically appropriate. This increased selection process will also decrease unnecessary exposure of healthcare personnel to potential asymptomatic COVID-19 patients with a false negative test secondary to airway management. Pulmonary resections for lung cancer require endotracheal intubation, as well as bronchoscopy for double lumen endotracheal tube placement. No report has described increased rate of COVID-19 transmission from thoracic surgery, regardless of exposure type, compared to other types of surgery.

All physicians and operating room personnel should screened daily for fever or development of other COVID-19 symptoms. Any provider who cared for patients positive for COVID-19 should undergo RT-PCR testing every month. Symptomatic health care providers should be tested for COVID-19. If positive, the provider should be quarantined for 2 weeks; if negative, the provider quarantined until asymptomatic for 5 days. Patients should also undergo COVID-19 RT-PCR testing within 72 h of the operation. If positive, we recommend 2 week quarantine followed by re-testing. During our experience from March 13th to May 4th, 2020, one (5%) of 21 patients was asymptomatic and found to be COVID-19 positive prior to surgery (30). Surgery was performed once the patient had a negative COVID-19 test. Regardless of the negative test, anesthesiologists should employ heightened intubation precautions. During intubation and bronchoscopy to position the endotracheal tube, all non-necessary personnel should wait outside the operating room. All surgical cases were performed through a minimally invasive approach (robotic or video-assisted) with the goal of safe expedited discharge for outpatient follow-up to minimize risk of in-hospital viral transmission.

Standard post-operative care regarding chest tube management and discharge criteria should be used, including specific precautions regarding superspreading procedures such as extubation, and peri-operative respiratory therapy such as nebulized bronchodilators, incentive spirometry, high-flow nasal cannula oxygen and non-invasive ventilation. All in-hospital care was provided on specific COVID free operating rooms, ICUs and floors. No patients could have visitors during their hospital stay and follow up after resection was done using telehealth visits. Patients with T1/T2 N0 NSCLC were reviewed in the multi-disciplinary and if stereotactic radiation was an option, it should be recommended and discussed with patients. In patients for whom surgery was delayed, consideration was given for neoadjuvant systemic chemotherapy.

### Changes to Radiation Therapy for Lung Cancer

Several changes in the clinical workflow should be made to meet adequate social distancing guidelines for lung cancer patients needing radiation. All follow-ups that do not require a physical exam should be changed to a virtual telemedicine platform. Most patients with stage I and II lung cancer considered for stereotactic body radiotherapy (SBRT) are elderly with poor lung function and other comorbidities that preclude surgical management; therefore, SBRT should be post-poned as appropriate during surge phase to minimize COVID-19 infection risk (31). Furthermore, we considered single fraction SBRT based on encouraging results from RTOG 0915 which demonstrated comparable local tumor control with 34Gy in a single fraction vs. 48Gy in four consecutive daily fractions (32). No restrictions need to be imposed on curative intent concurrent or sequential chemoradiation treatments for patients with locally advanced small cell lung cancer (SCLC) and NSCLC.

Given the lack of level I evidence and controversy surrounding post-operative radiotherapy for patents with fully resected N2 lung cancer (33), we recommend against adjuvant radiotherapy. Similarly, despite level I evidence supporting consolidative thoracic radiotherapy for patients with extensive stage small cell lung cancer (SCLC) (28), the lack of inclusion of thoracic RT in the IMPOWER 133 (34) and CASPIAN (35) studies combined with the poor prognosis and often compromised lung function of these patients led us to discontinue offering RT in this context. We also do not offer prophylactic cranial irradiation for patients with SCLC during the surge phase. Palliative radiotherapy was delivered with a single fraction for bone metastases when possible given similar initial efficacy to more protracted courses (36). Hypofractionation (17Gy in two fractions) was considered for patients requiring urgent palliative thoracic radiotherapy (37).

All patients should be evaluated for adequate pulmonary function, though this may be challenging as pulmonary function labs may be closed due to infection transmission concerns related to COVID-19. If recent pulmonary function tests are unavailable, we recommend clinical estimation of the pulmonary function using prior pulmonary function tests if available, baseline oxygen saturation, virtual walk desaturation testing and stair challenge. The standard radiation treatment time slots were increased from 15 to 20 min to minimize waiting room time as much as possible. All patients should masked, screened for COVID-19 symptoms, checked for elevated temperature, and maintained social distancing protocols. COVID-19 testing should be performed on all patients prior to CT simulation. Patients with active COVID-19 infection were treated with radiation therapy if the initiation or continuation of such therapy was deemed essential following MDTOC review. Such patients were typically scheduled for last patient of the day treatments followed by terminal room and facility cleans. With these protocols in place, we were able to continue offering radiotherapy without significant staff shortages necessitating forced patient delays throughout the pandemic.

## Conclusions

Management is different based on whether the hospital system is in surge phase and the capacity of the hospital. We have proven that lung cancer surgery can be safely performed in a hospital with census saturated with COVID-19 patients. Furthermore, we have had providers care for both COVID-19 patients and non-COVID-19 lung cancer patients, with a 0% transmission rate. It is not feasible to have dedicated non-COVID centers for oncologic surgery as it decreases available resources in a pandemic; therefore, the recommendations summarized in this review can guide cancer centers to continue to provide lung cancer care during COVID-19 pandemic with minimal added risk to patients and healthcare providers.

## Author Contributions

All authors listed have made a substantial, direct and intellectual contribution to the work, and approved it for publication.

## Conflict of Interest

The authors declare that the research was conducted in the absence of any commercial or financial relationships that could be construed as a potential conflict of interest.
